# Engineering immunity in osteosarcoma: nanomedicine strategies for overcoming immune evasion

**DOI:** 10.3389/fimmu.2026.1897106

**Published:** 2026-07-31

**Authors:** Qin Huang, Gongchang Yu, Bin Shi, Tingting Deng

**Affiliations:** 1 Neck-Shoulder and Lumbocrural Pain Hospital of Shandong First Medical University, Shandong First Medical University & Shandong Academy of Medical Sciences, Jinan, China; 2 College of Health and Elderly Care, Shandong Women’s University, Jinan, China

**Keywords:** immunosuppression, immunotherapy resistance, nanomedicine delivery systems, osteosarcoma, translational oncology, tumor microenvironment (TME)

## Abstract

Osteosarcoma is an aggressive malignancy characterized by inherent chemoresistance, a high propensity for pulmonary metastasis, and dismal survival rates in patients with advanced or refractory disease. While immunotherapy has revolutionized cancer treatment, its efficacy in osteosarcoma remains severely limited by an immunologically “cold” and profoundly immunosuppressive tumor microenvironment (TME). This TME is defined by pervasive infiltrates of tumor-associated macrophages (TAMs) and myeloid-derived suppressor cells (MDSCs), cancer-associated fibroblasts (CAFs), and a dense mineralized extracellular matrix (ECM) barrier that collectively exclude or exhaust cytotoxic T cells. We examine the intricate mechanisms driving this immunotherapy resistance at the cellular and molecular levels. We then explore how rationally designed nanotechnology platforms can overcome these barriers. Specifically, we review targeted nanomedicine delivery systems engineered to home to bone tissue and reshape the TME landscape via strategies including TAM reprogramming, MDSC depletion, ECM clearance to relieve physical resistance, and activation of innate immune pathways. Unlike general immunotherapy reviews, the main contribution of this mini-review is strictly focused on innovative nanomedicine delivery platforms—specifically biomimetic, stimuli-responsive, and bone-targeted systems. We dissect how these rationally engineered platforms overcome the unique physical and cellular barriers of the osteosarcoma TME, with a critical emphasis on the severe translational hurdles, such as protein corona formation and *in vivo* stability, that limit clinical progression. In conclusion, multi-targeted nanomedicine delivery systems hold strong preclinical potential to provide a novel framework for advancing osteosarcoma treatment by precisely modulating the TME and eliciting durable antitumor immune responses.

## 1 Introduction

Osteosarcoma is an aggressive malignancy with high metastatic potential and stagnant survival outcomes due to inherent chemoresistance (1–4). While immunotherapy has revolutionized the treatment of many solid tumors, its application to osteosarcoma has yielded modest clinical benefits, primarily because these tumors are immunologically “cold” (5–7). This immunotherapy resistance is driven by a highly immunosuppressive tumor microenvironment (TME) (7–9). The osteosarcoma TME is characterized by dense infiltrates of immunosuppressive cells, including tumor-associated macrophages (TAMs) and myeloid-derived suppressor cells (MDSCs), as well as physical barriers formed by cancer-associated fibroblasts (CAFs) and a dense extracellular matrix (ECM) that restrict cytotoxic T cell infiltration (10–12). The convergence of nanotechnology and immunotherapy offers a targeted approach to overcome these specific microenvironmental barriers (13–15).

The convergence of nanotechnology and immunotherapy offers a promising translational strategy to overcome these barriers. Nanomedicine platforms, engineered with precise control over size, surface chemistry, and drug release kinetics, can enhance the bioavailability and tumor-specific delivery of immunotherapeutic agents while minimizing systemic toxicity (13–15). More importantly, multifunctional nanoparticles can be designed to simultaneously modulate multiple components of the immunosuppressive TME, reprogram immune cell polarization, and synergize with conventional therapies to elicit durable antitumor immune responses (16–18). Bone-targeted nanocarriers, functionalized with moieties such as bisphosphonates or oligopeptides that bind hydroxyapatite, enable preferential accumulation within the bone TME, addressing a critical limitation of systemic immunotherapy delivery (19–21).

To distinguish from the broadly discussed literature on sarcoma nanomedicine, the main contribution of this mini-review is specifically focused on the precise intersection of engineered nanoplatforms and translational barriers. We first briefly outline the unique spatial and cellular immunosuppressive barriers in osteosarcoma. Importantly, we then critically evaluate how advanced delivery systems—particularly biomimetic, stimuli-responsive, and bone-targeted nanocarriers—are rationally engineered to dismantle these exact barriers. Finally, we highlight the often-overlooked translational hurdles, providing a focused roadmap for bringing nanomedicine-enabled immunotherapy into clinical practice.

## 2 The immunotherapeutic landscape of osteosarcoma: challenges and resistance mechanisms

### 2.1 The immunosuppressive TME

The osteosarcoma TME is a complex ecosystem comprising malignant cells, stromal cells, immune infiltrates, and ECM components embedded within a mineralized bone scaffold (9, 22). This microenvironment is profoundly immunosuppressive, creating multiple layers of resistance that limit the effectiveness of immunotherapeutic interventions. Unlike many adult epithelial cancers that respond to immune checkpoint blockade, osteosarcoma exhibit features of immune exclusion and immune desertification, where cytotoxic T cells are either absent from the tumor core or physically segregated from malignant cells (5, 23). Single-cell transcriptomic analyses have revealed that osteosarcoma tissues harbor a preponderance of immunosuppressive myeloid cells, dysfunctional T lymphocytes, and regulatory dendritic cells that collectively shape a milieu resistant to immune activation (6, 24).

TAMs represent the most abundant immune cell population within the osteosarcoma TME, often accounting for over 50% of infiltrating immune cells (25, 26). These macrophages are predominantly polarized toward the M2 phenotype, which secretes immunosuppressive cytokines such as IL-10 and TGF-β, promotes angiogenesis, and facilitates tumor cell invasion and metastasis (25–27). The balance between pro-inflammatory M1 and anti-inflammatory M2 macrophages is a critical determinant of antitumor immunity, and the dominance of M2-polarized TAMs in osteosarcoma correlates with poor prognosis and resistance to immune-based therapies (9, 28). Mechanistically, osteosarcoma cells actively drive M2 polarization through the secretion of extracellular vesicles, including migrasomes and exosomes, that carry immunomodulatory cargoes such as milk fat globule-EGF factor 8 (MFGE8), which enhances phagocytosis and promotes macrophage reprogramming toward a tumor-supportive phenotype (29, 30).

MDSCs constitute another major immunosuppressive population in the osteosarcoma TME. These heterogeneous cells are broadly classified into two major subsets: polymorphonuclear MDSCs (PMN-MDSCs) and monocytic MDSCs (M-MDSCs). While both subtypes potently inhibit effector T cells, they often emphasize distinct suppressive mechanisms; for instance, PMN-MDSCs predominantly utilize reactive oxygen species (ROS), whereas M-MDSCs rely more heavily on the production of nitric oxide (NO) and immunosuppressive cytokines. MDSCs expand in response to tumor-derived factors and suppress T cell function through multiple mechanisms, including the depletion of arginine and cysteine, production of ROS, and secretion of immunosuppressive cytokines (31–33). In osteosarcoma, MDSCs contribute to the failure of immune checkpoint inhibitors by creating a hostile microenvironment that prevents effective T cell activation and infiltration (7, 33). Targeting MDSCs has therefore emerged as a promising strategy to enhance immunotherapy efficacy, and nanomedicine platforms designed to deplete or reprogram these cells are under active investigation (31, 32).

CAFs represent a third critical component of the immunosuppressive TME. Activated CAFs secrete cytokines such as IL-6 and TGF-β, remodel the ECM to create physical barriers, and promote immune evasion through paracrine signaling (10, 12, 34). In osteosarcoma, a distinct subpopulation of CD36+ CAFs has been identified that internalizes oxidized low-density lipoprotein (OxLDL) and activates the PPARG-FABP4 metabolic axis, leading to secretion of ANGPTL4. This factor binds to integrins on CD8+ T cells and activates the JAK2-STAT3 pathway, driving T cell exhaustion and impairing effector function (35). The metabolic reprogramming of CAFs thus represents a novel mechanism of immunotherapy resistance that can be targeted using nanoparticle-based approaches (12, 35).

### 2.2 Immune evasion mechanisms at the cellular and molecular level

osteosarcoma employ multiple strategies to evade immune recognition and destruction. One prominent mechanism is the downregulation of major histocompatibility complex class I (MHC-I) molecules on tumor cells, which impairs antigen presentation and reduces the visibility of malignant cells to cytotoxic T lymphocytes (5, 24). In osteosarcoma, single-cell sequencing studies have demonstrated that MHC-I molecules are frequently downregulated, and this reduction in tumor immunogenicity constitutes a key mechanism of immune escape (24). Epigenetic silencing of antigen presentation machinery, including Polycomb-mediated repression and DNA hypermethylation at the HLA class I/NLRC5/interferon axis, further diminishes peptide display and limits T cell recognition (36).

The expression of immune checkpoint molecules, including PD-L1, provides another layer of immune evasion. PD-L1 is upregulated on osteosarcoma cells and interacts with PD-1 on activated T cells to induce exhaustion and apoptosis (37, 38). Thrombospondin-1 (TSP1) has been shown to upregulate PD-L1 expression in osteosarcoma through activation of the STAT3 pathway, thereby impairing antitumor immunity (37). While PD-1/PD-L1 blockade has demonstrated remarkable success in several cancer types, its efficacy in osteosarcoma remains limited, likely due to the redundant and interconnected nature of immunosuppressive pathways operating within the TME (6, 39). In addition to the classical PD-1/PD-L1 pathway, the T-cell immunoreceptor with Ig and ITIM domains (TIGIT)/CD155 axis has recently emerged as a critical mediator of immune evasion in osteosarcoma. CD155 (poliovirus receptor) is frequently overexpressed on osteosarcoma cells and interacts with TIGIT expressed on infiltrating cytotoxic T cells and natural killer (NK) cells. This interaction delivers potent inhibitory signals that drive effector cell exhaustion and facilitate tumor immune escape. Given the redundant nature of immunosuppressive pathways in the TME, co-targeting the TIGIT/CD155 axis alongside PD-1 blockade—potentially facilitated by multi-payload nanomedicine delivery systems—represents a highly promising frontier for overcoming resistance and restoring robust antitumor immunity.

**Figure 1:**
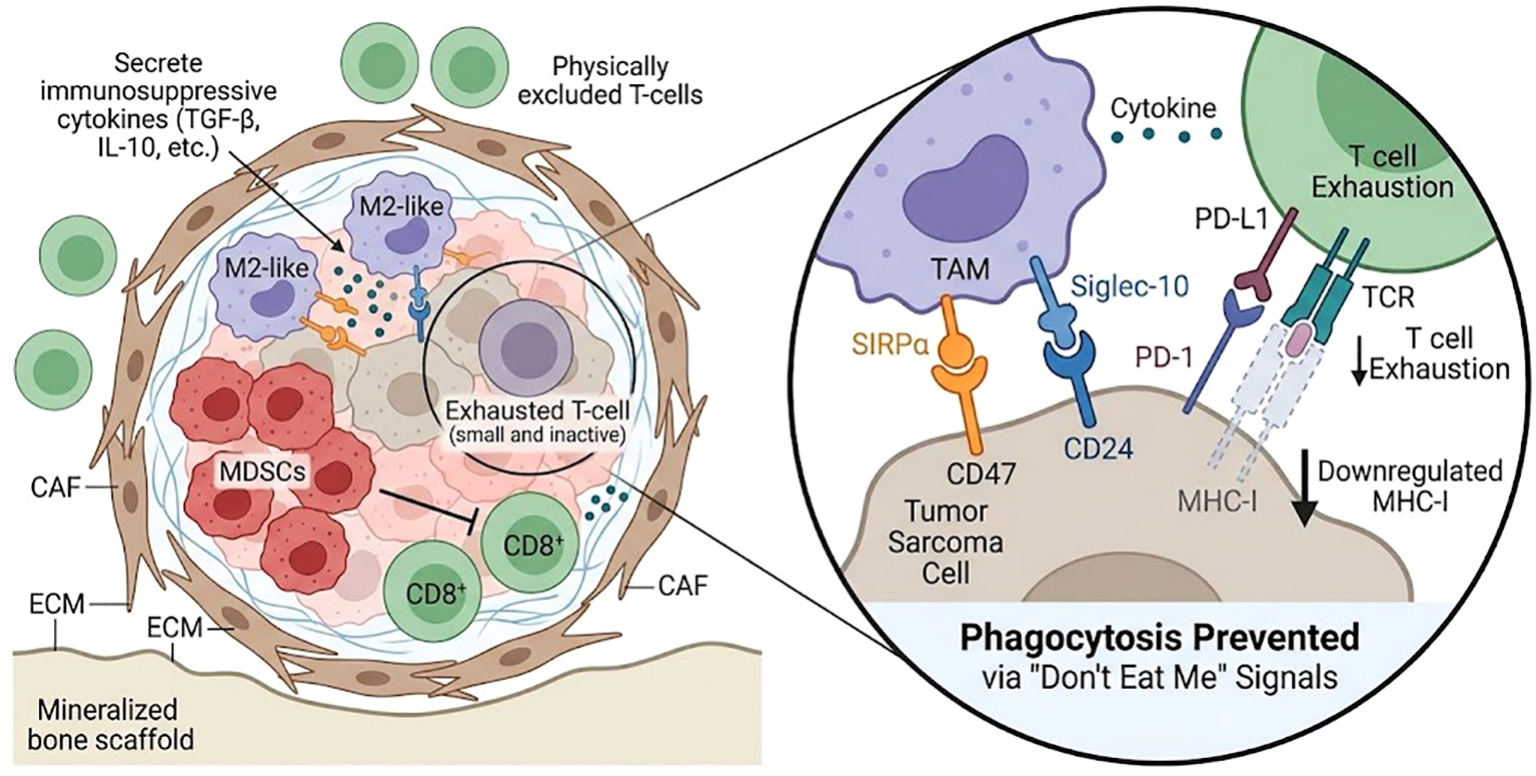
The immunosuppressive TME and mechanisms of immune evasion in osteosarcoma.

Beyond classical checkpoint pathways, osteosarcoma cells express “don’t eat me” signals that protect them from phagocytosis. CD24 has been identified as a novel immune evasion molecule in osteosarcoma that inhibits macrophage-mediated engulfment of tumor cells (24). Similarly, the CD47-SIRPα axis represents a critical phagocytosis checkpoint, and strategies to block this interaction using engineered nanoparticles have shown promise in restoring macrophage antitumor activity (40). The identification of these alternative immune checkpoints highlights the need for multi-targeted immunotherapeutic approaches that nanomedicine platforms are uniquely positioned to deliver (**Figure 1**).

The TME of osteosarcoma is characterized by a dense, mineralized extracellular matrix (ECM) and the infiltration of immunosuppressive cell populations that collectively orchestrate resistance to immunotherapy. (Left) At the cellular level, CAFs and the ECM form a physical barrier that restricts the infiltration of CD8+ cytotoxic T cells. TAMs, predominantly of the M2-like phenotype, and MDSCs secrete immunosuppressive cytokines (e.g., IL-10, TGF-β) that inhibit T cell function and drive immune exhaustion. (Right) At the molecular level, osteosarcoma cells utilize multiple immune checkpoints to evade immune clearance. Upregulation of the PD-L1/PD-1 axis induces T cell exhaustion, while the expression of “don’t eat me” signals, such as CD47 (interacting with SIRPα) and CD24 (interacting with Siglec-10), prevents macrophage-mediated phagocytosis. Furthermore, the downregulation of major histocompatibility complex class I (MHC-I) molecules impairs antigen presentation, effectively hiding tumor cells from cytotoxic T lymphocytes.

Furthermore, conventional chemotherapy resistance, a hallmark of osteosarcoma, actively exacerbates this immunosuppressive TME (41, 42). However, specific chemotherapeutic agents can induce immunogenic cell death (ICD), characterized by the release of damage-associated molecular patterns (DAMPs) that prime antitumor T cell responses (43, 44). The challenge of safely delivering these agents at sufficient doses to induce ICD without systemic toxicity highlights the necessity for spatiotemporally controlled nanomedicines, effectively bridging chemotherapy and immunotherapy (44–46).

## 3 Nanotechnology-enabled strategies to overcome immunosuppressive barriers

### 3.1 Reprogramming the tumor-associated macrophage compartment

Given the central role of M2-polarized TAMs in maintaining immunosuppression within the osteosarcoma TME, strategies to reprogram these cells toward a pro-inflammatory M1 phenotype have attracted considerable attention. Nanomedicine platforms offer precise tools for macrophage modulation, enabling the targeted delivery of polarization-inducing agents directly to the TME while sparing peripheral tissues (16, 18, 27). Mechanistically, the successful transition from a tumor-promoting M2 phenotype to a tumor-suppressive M1 state typically requires profound intracellular signaling shifts. This reprogramming is commonly characterized by the suppression of the STAT3 and STAT6 signaling cascades—which are essential for maintaining the immunosuppressive state (47–50)—accompanied by the robust activation of the NF-κB and STAT1 pathways that drive the transcription of pro-inflammatory cytokines and immunostimulatory factors (51, 52).

A multifunctional poly(amino acid) nanomedicine (PM-DPA/R848) has been developed that encapsulates the TLR7/8 agonist resiquimod (R848) within a nanocarrier composed of poly(L-methionine) and alendronate-functionalized DSPE-PEG (27). The poly(L-methionine) component reduces intracellular ROS levels in macrophages by 63.8%, leading to a 31.1% reduction in M2-polarized macrophages, while R848 release activates the MYD88/IRAK1/NF-κB pathway to drive M1 reprogramming, increasing the M1 macrophage population by 1.4-fold. The alendronate moiety enhances intratumoral accumulation, and the combined nanomedicine achieves a 77% tumor suppression rate in preclinical models (27).

Calcium carbonate (CaCO3)-based nanosystems have been engineered to simultaneously neutralize the acidic TME and reprogram TAM polarization. The A-NPs@(SHK+Ce6) nanosystem incorporates CaCO3 nanoparticles to buffer excess H+ ions and alleviate suppressive conditions, while the loaded SHK peptide inhibits lactate production and synergizes with photodynamic therapy to repolarize TAMs toward the M1 phenotype (53). When combined with PD-1 checkpoint blockade, this nanosystem demonstrates remarkable ability to eliminate distant tumors and promote long-term immune memory, highlighting the potential of multi-targeted nanoplatforms to overcome the spatial and functional heterogeneity of the TME (53).

Biomimetic approaches that leverage macrophage membranes for nanoparticle coating represent an emerging strategy to enhance targeting and immune modulation. Engineered M1 macrophage membranes overexpressing SIRPα have been used to encapsulate microwave-responsive nano-Prussian blue particles (SIRPα-M@nanoPB) (40). Upon microwave irradiation, these nanoparticles induce tumor cell death through hyperthermia and microwave dynamic effects, while the SIRPα on the nanoparticle surface actively binds CD47 on tumor cells, disrupting the “don’t eat me” signal and restoring macrophage phagocytosis. This dual-action approach not only directly eliminates tumor cells but also reprograms the immunosuppressive TME, reducing the risk of tumor recurrence following local ablation (40). However, a major limitation of macrophage-reprogramming strategies lies in the extreme plasticity of TAMs and high tumor heterogeneity, which can cause reprogrammed M1 macrophages to revert to an M2 state. Furthermore, potent immunomodulators risk off-target immune activation and systemic cytokine storms if nanocarrier biodistribution is suboptimal (27, 54).

### 3.2 Targeting MDSCs

Depleting or functionally inactivating MDSCs represents a complementary strategy to enhance antitumor immunity in osteosarcoma. Nanomedicine platforms can be designed to selectively target MDSCs based on their surface receptor expression or metabolic vulnerabilities, thereby reducing systemic immunosuppression while preserving beneficial myeloid populations (32, 33).

A hyaluronic acid (HA)-modified metal-organic framework (MOF) has been developed for combined chemotherapy and immunotherapy of osteosarcoma (31). This nanosystem co-delivers gemcitabine, which depletes MDSCs, and D-1-methyltryptophan, an inhibitor of indoleamine 2,3-dioxygenase (IDO), an enzyme that promotes T cell suppression. The HA modification enables CD44-mediated targeting of tumor cells, while the ZIF-8 framework provides pH-responsive drug release within the acidic TME. In preclinical models, this nanoplatform effectively reduces MDSC populations, reactivates antitumor immunity, and inhibits tumor growth with a favorable safety profile (31).

Adoptive cellular therapy using engineered macrophages represents another innovative approach to overcome MDSC-mediated immunosuppression. Pexidartinib-loaded ZIF-8 nanoparticles (P@ZIF-8) have been loaded into M1 macrophages decorated with K7M2-targeting peptides (P@ZIF/M1-KTP) (55). These engineered macrophages home to tumor sites, release pexidartinib to inhibit CSF-1 receptor signaling and reduce MDSC accumulation, and themselves contribute to antitumor immunity through their M1 phenotype. Treatment with P@ZIF/M1-KTP significantly increases the ratio of CD4+ T cells and decreases MDSCs in tumor tissues, demonstrating the potential of combining nanoparticle drug delivery with cellular therapy to remodel the immunosuppressive TME (55). Despite these advances, systemic MDSC depletion carries the risk of off-target toxicity to normal myeloid cell differentiation. Ensuring absolute targeting specificity to the TME to preserve peripheral immunity remains a critical biodistribution challenge for these platforms (32).

### 3.3 Modulating CAFs and ECM

CAFs contribute to immunotherapy resistance through multiple mechanisms, including physical barrier formation, secretion of immunosuppressive cytokines, and metabolic reprogramming of the TME (10, 12, 34). Nanomedicine strategies targeting CAFs aim to either deplete these cells, inhibit their pro-tumorigenic functions, or reprogram them toward a tumor-restraining phenotype.

The identification of CD36+ CAFs as a key driver of immunosuppression in osteosarcoma has opened new avenues for targeted intervention (35). These CAFs uptake OxLDL and activate the PPARG-FABP4 axis, leading to ANGPTL4 secretion that promotes CD8+ T cell exhaustion. Administration of vitamin E (VitE), which scavenges OxLDL, effectively reprograms the TME by reducing CAF-mediated immunosuppression and synergizes with PD-1 blockade to improve tumor control (35). Nanoparticle-based delivery of VitE or other OxLDL scavengers could enhance the specificity and efficacy of this approach, enabling sustained modulation of CAF metabolism within the TME.

The dense ECM characteristic of osteosarcoma presents a physical barrier that restricts T cell infiltration and limits the penetration of therapeutic agents (11, 12). Photothermal-triggered ECM clearance using mesoporous polydopamine nanoparticles encapsulating bromelain and the immune adjuvant R848 (M@B/R) has been developed to address this limitation (11). Upon near-infrared irradiation, photothermal therapy induces ICD and activates dendritic cell maturation, while the heat-triggered release of bromelain degrades collagen fibers within the ECM, facilitating cytotoxic T lymphocyte penetration into the tumor core. This integrated approach overcomes the physical barriers imposed by ECM-rich tumors and significantly enhances antitumor immunity in preclinical osteosarcoma models (11). Nevertheless, degrading the ECM presents a double-edged sword: if the localized tumor destruction is incomplete, the loosened matrix could paradoxically facilitate the escape of surviving malignant cells. Consequently, robust validation in clinically relevant orthotopic and metastatic osteosarcoma models, rather than simple subcutaneous models, is urgently required for these approaches (10–12).

### 3.4 Activating innate immune pathways through nanocarrier design

The cGAS-STING pathway has emerged as a critical node for activating innate antitumor immunity, and its stimulation has shown promise in converting immunologically cold tumors into hot ones (21, 56). Nanotechnology-based approaches to activate cGAS/STING signaling offer the advantage of targeted delivery and sustained activation while minimizing systemic inflammatory toxicity.

A bone-targeted, glutathione-responsive polymeric nanoparticle (NPALN/Mn-AP) has been engineered to chelate manganese (Mn) and deliver inhibitors of ATM and PRMT5 (21). By functionalizing the nanoplatform with alendronate, preferential accumulation in bone tumors is achieved. Upon cellular uptake, elevated intracellular glutathione levels trigger the controlled release of both inhibitors, which amplify DNA damage and activate the cGAS-STING pathway. The Mn ions further enhance this innate immune signaling by promoting cytosolic DNA sensing, leading to reshaping of the TME toward a more immune-responsive state and robust antitumor immunity in osteosarcoma models (21).

**Figure 2:**
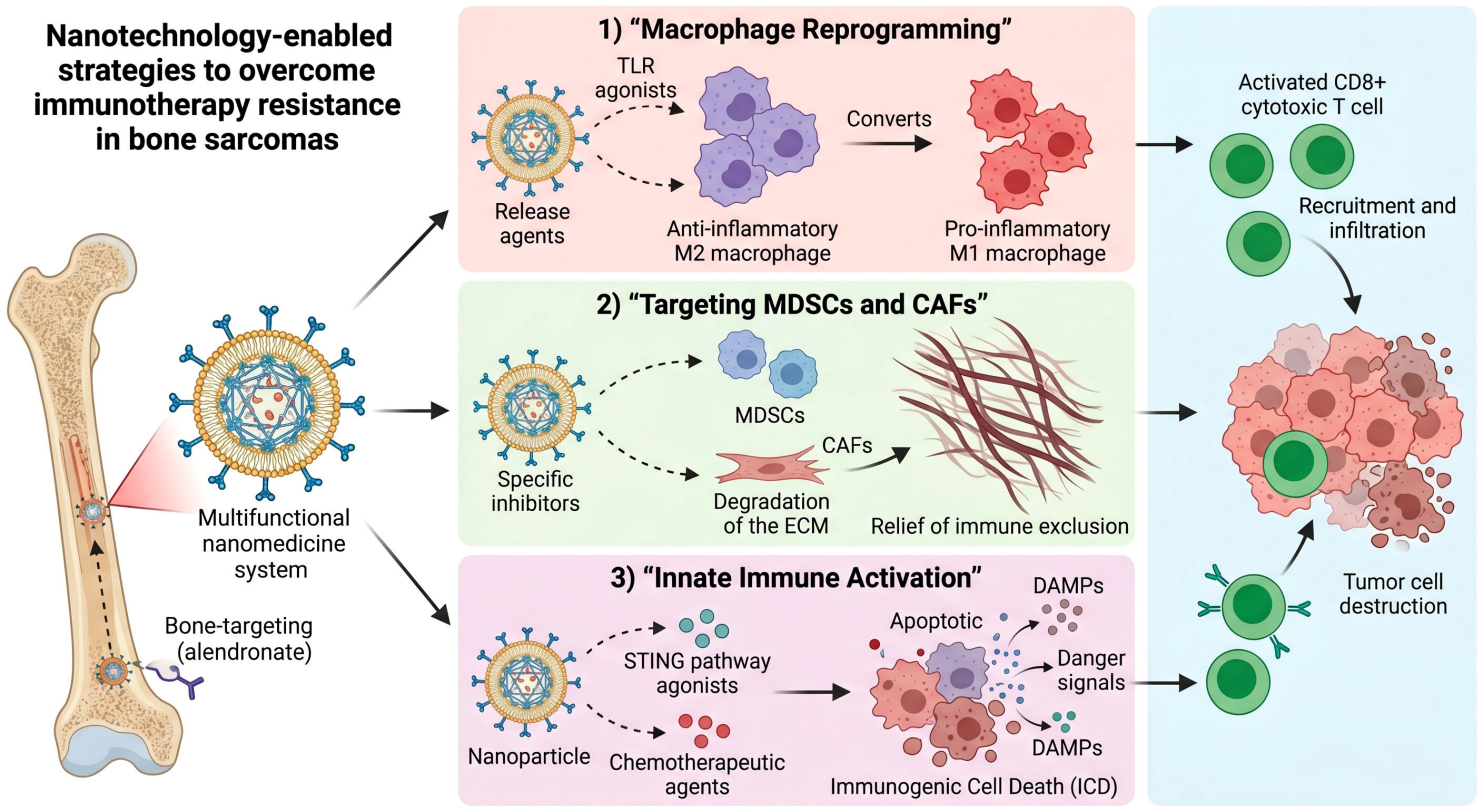
Nanotechnology-enabled strategies to overcome immunotherapy resistance in the osteosarcoma TME.

Copper-based nanomaterials represent another class of innate immune activators. Cu2-xSe nanoparticles encapsulated within PLGA-PEG-iRGD (Cu2-xSe(R837)-iRGD) have been shown to induce cuproptosis, inhibit tumor cell proliferation, and promote ICD (57). When combined with radiotherapy and PD-1 blockade, these nanoparticles promote CD8+ T cell infiltration and activate antitumor immune responses, demonstrating the potential of metal-based nanoplatforms to synergize with multiple therapeutic modalities (57) (**Figure 2**). A significant translational barrier for these metal-based (Mn, Cu) innate immune activators is the potential for long-term heavy metal toxicity. The rigorous evaluation of their *in vivo* clearance, off-target accumulation, and auto-inflammatory side effects is mandatory prior to clinical application (21, 56).

Multifunctional nanomedicine delivery systems can be rationally designed to target and reprogram the immunosuppressive milieu of osteosarcoma. (Left) Nanoparticles functionalized with bone-targeting moieties, such as alendronate, enable preferential accumulation within the mineralized bone scaffold. (Middle) Upon reaching the tumor site, these nanocarriers exert their therapeutic effects through three primary mechanisms (1): Macrophage Reprogramming, where the delivery of immunomodulators (e.g., TLR agonists) converts tumor-promoting, anti-inflammatory M2 macrophages into tumor-suppressive, pro-inflammatory M1 macrophages; (2) Targeting MDSCs and CAFs, in which specific inhibitors deplete immunosuppressive myeloid cells and degrade the dense ECM to relieve T cell immune exclusion; and (3) Innate Immune Activation, where the release of STING pathway agonists or chemotherapeutic agents triggers ICD, leading to the release of DAMPs and danger signals. (Right) Collectively, these nanomedicine-driven interventions reshape the TME, facilitating the robust recruitment, infiltration, and activation of CD8+ cytotoxic T cells to achieve effective tumor cell destruction.

## 4 Translational applications and innovative delivery platforms in preclinical models

### 4.1 Biomimetic and cell membrane-coated nanoparticles

The clinical translation of nanomedicine platforms for osteosarcoma immunotherapy requires not only potent antitumor activity but also favorable biocompatibility, targeted delivery, and the ability to overcome biological barriers. Biomimetic nanoparticles, which leverage natural cell membranes for surface coating, have emerged as a promising strategy to achieve these goals (40, 54, 58).

A biomimetic co-delivery system engineered from genomic insights has been developed for precision treatment of osteosarcoma (58). Based on the identification of CDK4 amplification and homologous recombination repair deficiency as prominent features of osteosarcoma, a MOF co-loaded with CDK4/6 inhibitors and PARP inhibitors was designed and coated with a bio-membrane targeting B7H3. This targeted biomimetic system precisely homes to osteosarcoma cells with high B7H3 expression, and the synergistic combination of CDK4/6 and PARP inhibition severely impairs the tumor’s DNA repair capacity. Furthermore, the system activates the immune microenvironment by increasing CD8+ T cell infiltration, converting osteosarcoma from an immune-cold to an immune-hot tumor (58).

Cell membrane-coated nanoparticles offer additional advantages for immune evasion and prolonged circulation. By cloaking synthetic nanoparticle cores with membranes derived from immune cells, these platforms inherit the surface receptor repertoire of the source cells, enabling them to evade immune clearance and actively target tumor tissues (54). Macrophage membrane-coated nanoparticles, in particular, have shown promise for delivering immunomodulatory cargoes to the TME while avoiding recognition by the reticuloendothelial system (40, 54).

### 4.2 Stimuli-responsive nanoplatforms for spatiotemporal drug release

The TME of osteosarcoma exhibits distinct physicochemical features, including acidic pH, elevated glutathione levels, and hypoxia, that can be exploited for stimuli-responsive drug release (13, 45, 59). These features enable the design of nanocarriers that remain stable in circulation but rapidly release their payloads upon reaching the tumor site, maximizing therapeutic efficacy while minimizing off-target toxicity.

A hierarchical MOF has been engineered for spatiotemporal control of immunotherapy and chemotherapy in osteosarcoma (46). This nanosystem is armed with PD-L1 antibody on its surface and loaded with C-C motif chemokine ligand 19 (CCL19) and oxaliplatin in separate layers. The anti-PD-L1 serves dual functions: it targets the nanoparticles to PD-L1-expressing tumor cells and simultaneously blocks the PD-1/PD-L1 interaction to prevent T cell exhaustion. The initial release of CCL19 recruits additional T cells into the TME, while the pH-responsive release of oxaliplatin upon cellular uptake induces maximum cytotoxicity. This programmable action schedule enables each drug to exert its optimal effect without mutual interference, resulting in a tumor suppression rate exceeding 90% with minimal side effects (46).

An autophagy-controlling nanocarrier (CUR-BMS1166@ZIF-8@PEG-FA, CBZP) has been designed to enhance PD-1/PD-L1 blockade immunotherapy in osteosarcoma (44). This pH-sensitive nanoparticle loads curcumin to activate ICD via autophagic cell death and BMS1166 to simultaneously inhibit the PD-1/PD-L1 interaction. The pH-sensitive ZIF-8 framework induces autophagy and decreases intracellular pH, which in turn facilitates the release of curcumin to further enhance autophagic activity. In orthotopic osteosarcoma models, CBZP demonstrates powerful antitumor effects and establishes long-term immunity against tumor recurrence, accompanied by enhanced dendritic cell maturation and tumor infiltration of CD8+ T lymphocytes (44).

### 4.3 Bone-targeted delivery systems

Efficient delivery of therapeutic agents to bone tumors is complicated by the unique physiological features of bone tissue, including its dense mineralized matrix, low blood flow in certain regions, and the presence of the bone marrow niche (19, 20, 59). Bone-targeted nanocarriers functionalized with moieties that bind to hydroxyapatite, the primary mineral component of bone, have been developed to overcome these barriers (19–21).

Alendronate, a bisphosphonate with high affinity for bone mineral, has been widely used for bone-targeted drug delivery. Conjugation of alendronate to nanoparticle surfaces enables preferential accumulation at bone tumor sites, as demonstrated in multiple preclinical studies (19, 21, 27). The NPALN/Mn-AP system described earlier utilizes alendronate functionalization to achieve bone-targeted delivery of ATM and PRMT5 inhibitors along with Mn ions for cGAS-STING activation (21). Similarly, the PM-DPA/R848 nanomedicine incorporates alendronate-containing DSPE-PEG-ALN to enhance intratumoral drug accumulation (27).

IL-11Rα represents another promising target for bone-specific drug delivery. IL-11Rα is highly expressed in osteosarcoma tissues, and peptide ligands targeting this receptor have been used to functionalize nanocarriers for enhanced tumor specificity (45, 60). IL-11Rα-targeted polymersomes encapsulating doxorubicin (IL11-PDox) demonstrated significantly augmented internalization and apoptotic activity in IL-11Rα-overexpressing osteosarcoma cells, leading to striking repression of tumor growth and lung metastasis in orthotopic and relapsed models (60). An injectable TME-responsive nanocomposite hydrogel incorporating IL11Rα-targeted liposomes has also been developed for local delivery of cisplatin-loaded MnO2 along with a MYC inhibitor, enabling sustained drug release and reshaping of the immune microenvironment in MYC-amplified osteosarcoma (45).

### 4.4 Combination strategies: nanomedicine-enhanced chemo-immunotherapy

The most promising applications of nanomedicine in osteosarcoma immunotherapy involve combination strategies that simultaneously target multiple resistance mechanisms. By co-delivering chemotherapeutic agents that induce ICD with immunomodulators that reprogram the TME, these platforms can achieve synergistic antitumor effects that are not attainable with either modality alone (16, 43, 61).

The mechanotherapeutic agent ketotifen has been shown to reprogram the TME by reducing tumor stiffness and increasing perfusion, thereby enhancing the delivery and efficacy of nanomedicine-based chemo-immunotherapy (61). In fibrosarcoma and osteosarcoma models, ketotifen combined with pegylated liposomal doxorubicin (PLD) or pegylated liposomal coencapsulated alendronate-doxorubicin (PLAD) plus anti-PD-1 antibody improved antitumor responses, increased cytotoxic CD8+ T cell and CD4+ T helper cell infiltration, and Treg numbers. Additionally, the combination altered TAM polarization toward the M1 immune-supportive phenotype, demonstrating that TME reprogramming can significantly enhance the efficacy of nanomedicine-based immunotherapy (61).

Nanocarrier-based epirubicin micelles (NC-6300) have demonstrated anti-tumor activity in sarcoma patients, and their combination with anti-PD-L1 antibody has been extensively studied in preclinical models (43). NC-6300 induces ICD, and its effect on the efficacy of anti-PD-L1 antibody is dependent on T cells. Importantly, NC-6300 normalizes the TME by increasing blood vessel maturity and reducing fibrosis, leading to increased intratumor density and proliferation of T cells when combined with checkpoint inhibition. These findings suggest that nanocarrier-based chemotherapy can potentiate immune checkpoint inhibition by normalizing the TME, providing a mechanistic rationale for clinical translation (43).

### 4.5 Overcoming drug resistance through nanocarrier-mediated gene therapy

Non-coding RNAs, including miRNAs, long non-coding RNAs (lncRNAs), and circular RNAs (circRNAs), play critical roles in the development of chemoresistance in osteosarcoma, and their modulation using nanoparticle delivery systems represents a novel therapeutic approach (62–64). The competitive endogenous RNA (ceRNA) network involving circRNAs, lncRNAs, and miRNAs regulates the expression of genes involved in drug efflux, DNA repair, and apoptosis, and disruption of this network can sensitize resistant tumor cells to chemotherapy (62).

Nanoparticle-based delivery of tumor-suppressive miRNAs has shown promise in overcoming drug resistance in osteosarcoma (64). Polymer-based, lipid-based, and inorganic nanoparticles, as well as extracellular vesicles, have been used to deliver miRNAs that target key resistance pathways. These delivery systems can be modified to enhance drug loading and delivery capabilities, and miRNA delivery can be combined with conventional chemotherapy to achieve synergistic effects (64). The development of exosome-based delivery platforms is particularly attractive, as exosomes exhibit excellent biocompatibility, low immunogenicity, and the ability to cross biological barriers, making them ideal nanocarriers for gene therapy in osteosarcoma (65–67).

### 4.6 Overcoming translational barriers: *In Vivo* stability and manufacturing hurdles

Despite the immense preclinical promise of nanomedicine in osteosarcoma, several significant translational barriers must be addressed before clinical implementation (59, 68). Upon intravenous administration, nanoparticles are highly susceptible to protein corona formation, which can mask targeting ligands and trigger rapid opsonization (69, 70). Consequently, a significant proportion of administered nanocarriers is prematurely sequestered and cleared by the reticuloendothelial system (RES)—particularly the liver and spleen—substantially limiting the effective dose that reaches the osteosarcoma TME (69, 70). Balancing the structural robustness required to prevent premature drug leakage in systemic circulation with the necessity for stimuli-responsive disassembly at the tumor site remains a critical engineering challenge. Furthermore, scalability and manufacturing reproducibility represent major hurdles (59, 68). The complex, multi-component nature of targeted and stimuli-responsive nanoplatforms complicates large-scale production, rigorous quality control, and the evaluation of long-term biocompatibility for novel biomaterials and metal-based nanoparticles (13, 59, 68). Finally, the overwhelming reliance on ectopic subcutaneous models in current research fails to recapitulate the authentic bone microenvironment; future validations must strictly utilize clinically relevant orthotopic and metastatic osteosarcoma models to truly assess nanomedicine efficacy and biodistribution. A detailed comparison of these advanced nanomedicine platforms, including their immune mechanisms and translational limitations, is summarized in **Table 1**.

**Table 1:** Summary of nanomedicine platforms for osteosarcoma immunotherapy.

Nanoparticle type	Targeting ligand	Therapeutic cargo	Immune mechanism	Model used	Major outcome	Translational limitation	Ref
Poly(amino acid) nanomedicine	Alendronate (Bone-targeting)	R848 (TLR7/8 agonist)	Activates MYD88/IRAK1/NF-κB pathway; reduces ROS to drive M1 macrophage reprogramming	Preclinical osteosarcoma model	77% tumor suppression; 1.4-fold increase in M1 macrophages	Potential premature drug leakage; RES clearance before reaching bone	(27)
Calcium carbonate (CaCO3) nanoparticles	None explicitly (Acidity-responsive)	SHK peptide & Ce6 (Photosensitizer)	Neutralizes acidic TME; inhibits lactate production; repolarizes TAMs toward M1 phenotype	Distant tumor models	Eliminates distant tumors; promotes long-term immune memory with PD-1 blockade	Limited tissue penetration depth for photodynamic therapy (Ce6) activation	(53)
Macrophage membrane-coated Nano-Prussian blue	SIRPα (overexpressed on membrane)	Nano-Prussian blue	SIRPα binds CD47 to disrupt the “don’t eat me” signal; restores macrophage phagocytosis	Osteosarcoma model	Hyperthermia-induced tumor cell death; reduced risk of local recurrence	High complexity in cell membrane sourcing, standardization, and scale-up manufacturing	(40)
Metal-organic framework (MOF; ZIF-8)	Hyaluronic acid (HA) (Targets CD44)	Gemcitabine & D-1-methyltryptophan (IDO inhibitor)	Gemcitabine depletes MDSCs; D-1-methyltryptophan prevents IDO-mediated T cell suppression	Preclinical osteosarcoma model	Reduces MDSCs; reactivates antitumor immunity; favorable safety	MOF long-term biocompatibility and complex multi-drug loading scalability	(31)
Glutathione-responsive polymeric nanoparticle	Alendronate (Bone-targeting)	Manganese (Mn), ATM inhibitor, PRMT5 inhibitor	Amplifies DNA damage; Mn ions enhance cytosolic DNA sensing to activate cGAS-STING pathway	Osteosarcoma model	Reshapes the TME into an immune-responsive state; robust antitumor immunity	Potential heavy metal (Mn) toxicity and protein corona masking targeting ligands	(21)
Autophagy-controlling nanocarrier (CBZP)	Folic acid (FA)	Curcumin & BMS1166 (PD-1/PD-L1 inhibitor)	Induces autophagic immunogenic cell death (ICD); simultaneously inhibits PD-1/PD-L1 interaction	Orthotopic osteosarcoma model	Long-term immunity against recurrence; enhanced DC maturation and CD8+ T cell infiltration	In vivo stability of the pH-sensitive framework during systemic circulation	(44)

## 5 Conclusion and outlook

The integration of rationally engineered nanomedicine with immunotherapy offers a highly rational, albeit early-stage, approach to overcome the unique physical and cellular resistance mechanisms inherent to the osteosarcoma TME. By deploying biomimetic, stimuli-responsive, and bone-targeted nanoplatforms, researchers can precisely modulate TAM polarization, deplete MDSCs, and activate innate immune pathways while minimizing systemic toxicity. Moving forward, the most realistic near-term translational path lies in combining relatively simple, biocompatible bone-targeted nanocarriers (such as alendronate-functionalized liposomal or polymeric systems) to specifically co-deliver immunogenic cell death (ICD)-inducing standard chemotherapeutics alongside established immune checkpoint inhibitors (e.g., anti-PD-1/PD-L1) or myeloid cell modulators. It is crucial to explicitly note that the evidence presented herein remains overwhelmingly confined to preclinical models. While early-generation nanomedicines (such as liposomal mifamurtide) have historical clinical applications in osteosarcoma, and some nanocarriers like epirubicin micelles (NC-6300) have entered early-phase trials for general sarcomas, none of the advanced, multi-targeted nanomedicine-immunotherapy combination platforms discussed in this review have yet entered active clinical trials specifically for osteosarcoma. To bridge this gap, the field must pivot from proof-of-concept studies to rigorous translational validation. The key preclinical data urgently needed before clinical trials can commence include: (i) efficacy and immune-profiling data derived strictly from clinically relevant orthotopic and spontaneous pulmonary metastasis models, rather than simple ectopic subcutaneous models; (ii) comprehensive pharmacokinetic, biodistribution, and long-term toxicology profiles validated in large-animal models (e.g., canine osteosarcoma, which closely mimics human disease); and (iii) standardized, reproducible scale-up manufacturing protocols that meet Good Manufacturing Practice (GMP) standards. Only by achieving these strict preclinical milestones can nanomedicine safely and effectively transition from the bench to the bedside for patients with refractory osteosarcoma.
